# The Book World of Medicine and Science

**Published:** 1898-12-17

**Authors:** 


### The Book World of Medicine and Science.

Disinfection and Disinfectants. By S. Rideal, D.Sc. Lond. Second edition. Pp. 372. Illustrated. (London: The Sanitary Publishing Company, Limited. 1898. Price 12s. 6d.)

This is a book which summarises, critically and systematically, the literature, scattered through English and foreign publications, relating to disinfection. Its importance, therefore, to public officials, and to medical officers of health in particular, is very considerable. At the outset mechanical disinfection and disinfection by heat are discussed thoroughly, and almost every apparatus of importance figured and described. Particular attention is paid to the conditions of public installations. But, very naturally, the greater part of the book is given up to chemical disinfection, and we doubt if anywhere the subject has received more exhaustive and impartial treatment. Dr. Rideal's distinction as a chemist is sufficient guarantee of the accuracy of the purely chemical information furnished. Besides the systematic discussion of chemical disinfection, there are admirable chapters on the practical application, in particular cases, of the various processes. The preservation of food receives adequate mention, and there is a lucid *résumé* of legal enactments. Since the appearance of the first edition much information has been added in appendices—some sixty pages in all—concerning new inventions and chemical processes. The treatment of sewage—except from the point of view of disinfection in the strict sense—forms no part of Dr. Rideal's book. But it is impossible to express anything save admiration for the lucidity, care, and critical acumen with which Dr. Rideal has dealt with the vast amount of information summarised in these pages. The book is naturally not an easy one to read, references and citations abound, and doubtless any style other than an arid one would, under the circumstances, be out of place. But it is a vast magazine of facts, and, so far as we have been able to see, of almost unerring accuracy. There is a good index, and a bibliography, in addition to the many references given in the text and in footnotes.

Practical Uranalysis and Urinary Diagnosis. By C. W. Purdy, M.D., LL.D. Pp. 365, 8vo. Illus. Fourth Edition. Price $2.50. Philadelphia: The F. A. Davis Co. 1898. (London: Sampson Low, Marston, and Co.)

The plan of this handsome and well got-up book is one which, though involving not a little repetition, is of considerable advantage to the student. Part I.—Uranalysis—gives an account of normal urine and of the methods of analysis employed in detecting and estimating both normal and morbid constituents. Short paragraphs are inserted in appropriate places stating concisely the clinical importance of the different phenomena and pathological manifestations. Part II.—Urinary Diagnosis—is devoted to a systematic account of diseases—both "surgical" and "medical"—of the urinary organs, and especial stress is laid on the methods employed in their detection and differentiation. There is so much that is excellent in Dr. Purdy's book that we are forced to notice some of its shortcomings and omissions. In no department of science can omissions be secured against; but in a book of this character they should be few. We have found no mention, either in the index or text, of alkaptone and alkaptonuria; pyrocatechin and its derivatives are practically ignored. Bence-Jones' name finds no place in Dr. Purdy's pages, and the allusion to his famous "albumin" is so slight that the characteristic appearance of urine containing it in bulk—after cooling and before treatment with reagents—is not mentioned. The smegma bacillus surely ought not to escape notice in a book dealing with the bacteriology of urine. Indeed, the account of parasites generally is inadequate. In cases of hæmaturia from Bilharzia, not only the ova, but the embryos may be found in the urine. The account of filaria is very poor. The clinical relationship between hæmoglobinuria, Raynaud's disease, and congenital syphilis has escaped the author's notice, and we are surprised to find under the heading "Uræmia" no allusion to the symptoms of "obstructive suppression." But no account of uræmia can be considered adequate that ignores the remarkable work of Dr. Bradford. The author's modification of Dr. Pavy's ammoniated solution for estimating glycosuria is ingenious. The substitution of glycerine for sodic tartrate is doubtless of real value. But it is much more convenient, practically, to employ a solution of which 10 c.c.'s are reduced by ·05 grams grape sugar than one in which ·02 grams destroys the colour of 35 c.c.'s. The diagrams throughout the book are clear and well chosen, the author's style free from mannerisms, and a special word of praise is due to the publishers for the care and taste expended on the paper, printing, and binding of what makes a handsome and, we trust, a useful book.

Drs Harvey and Davidson's Syllabus of Materia Medica. Revised by W. Martindale, F.C.S., F.L.S. Tenth Edition. Pp. 64, 16mo. Price 1s. net. (London: H. K. Lewis.)

This little book has been known for years to students in the agony of preparation for their "materia medica" as one of the best aids to memory in getting up tables of preparations and doses. It has in this, the tenth edition, been revised by Mr. Martindale in accordance with the British Pharmacopœia of 1898. The original and ingenious plan of indicating by symbols the relative value of drugs, &c., has been retained, and, very rightly, tables of the metric system and approximate equivalents in it of official proportions have been added.

La Pratique des Maladies des Enfants dans les Hôpitaux de Paris. Par Le Professeur Paul Lefert. Second edition; entirely remodelled. (Paris: J. B. Baillière et Fils. 1898.)

This is an admirable little book, for, although it is but small, it offers recent and authoritative information on a multitude of subjects which are of daily interest to the practitioner. It is, in effect, a dictionary of treatment dealing with the diseases of children. The various subjects dealt with are arranged in alphabetical order, and the name of the author is in each case given. These authors are physicians and surgeons attached to the Parisian hospitals, and thus the book presents the actual practice in vogue at the present time. Some of the diseases are treated by only one writer, but under the headings of the more important maladies and those about which there may be any difference of opinion articles appear by many different men, so that the book becomes a symposium as well as a dictionary. Diphtheria, for example, is treated of by nine authorities, typhoid fever by eight, and whooping cough by ten. Thus a wide view is obtained of the treatment most in use, although, no doubt, the plan adopted involves some little repetition.

